# A *Plasmodium* plasma membrane reporter reveals membrane dynamics by live-cell microscopy

**DOI:** 10.1038/s41598-017-09569-4

**Published:** 2017-08-29

**Authors:** Paul-Christian Burda, Marco Schaffner, Gesine Kaiser, Magali Roques, Benoît Zuber, Volker T. Heussler

**Affiliations:** 1 Institute of Cell Biology, University of Bern, 3012 Bern, Switzerland; 2 Institute of Anatomy, University of Bern, 3012 Bern, Switzerland; 3 Present Address: Bernhard Nocht Institute for Tropical Medicine, 20359 Hamburg, Germany

## Abstract

During asexual replication within the *Anopheles* mosquito and their vertebrate host, *Plasmodium* parasites depend on the generation of a massive amount of new plasma membrane to produce thousands of daughter parasites. How the parasite plasma membrane (PPM) is formed has mostly been studied by electron microscopy, which does not allow an insight into the dynamics of this process. We generated a *Plasmodium berghei* reporter parasite line by GFP-tagging of a non-essential PPM-localized protein, and followed plasma membrane development in living parasites through the entire *Plasmodium* life cycle. By generating double-fluorescent parasites in which the PPM is visualized in combination with the parasite endoplasmic reticulum, we show that membrane contact sites are formed between both membrane systems during oocyst and liver stage development that might be used to deliver lipids to the dramatically expanding PPM. In conclusion, we have established a powerful tool to follow PPM development in living parasites, which promises to greatly expand our knowledge of membrane biology in the *Plasmodium* parasite.

## Introduction

Malaria remains one of the major global health burdens, continuing to threaten the life of approximately 40% of the world population. It is caused by *Plasmodium* parasites, which infect more than 200 million people per year, leading to about 430,000 deaths^1^. With no commercially available vaccine and increasing reports of drug resistances, there is an urgent need to understand the biology of the parasite in more detail in order to develop new parasite-specific intervention strategies.

Infection of mammals with *Plasmodium* parasites is initiated when sporozoites are transferred during a blood meal of an infected female *Anopheles* mosquito. Sporozoites are deposited under the skin of the host and subsequently travel to the liver, where they infect hepatocytes and undergo massive multiplication. From here, parasites are released into the blood, where they multiply in red blood cells, causing the symptoms of malaria. Some of the parasites differentiate into gametocytes, the sexual forms of the parasite. After these have been taken up by a mosquito during blood feeding, female and male gametes fuse within the mosquito midgut, resulting in the formation of a zygote that develops further into a motile ookinete. The ookinete traverses the midgut epithelium and differentiates into an oocyst, in which another round of multiplication occurs, resulting in the generation of sporozoites. After their release from oocysts, sporozoites migrate to the salivary glands of the mosquito, from where they can be injected into a new host to initiate another parasite life cycle (reviewed in ref. 2).

Each of the asexual replication steps in the *Plasmodium* life cycle is characterized by repeated rounds of nuclear division (schizogony), followed by cytokinesis to form new daughter cells, and is dependent on an enormous amount of membrane material. Not only do *Plasmodium* parasites need to extend the parasite plasma membrane (PPM) and to replicate their organelles during daughter cell formation, they also need to support the growth of the parasitophorous vacuole membrane (PVM), which surrounds parasites during their multiplication within hepatocytes and erythrocytes. How the high demand of lipid material needed for membrane biogenesis is met and how these lipids are then distributed in the parasite, is only partially understood.

Apart from the acquisition of lipid material, parasites also have to control the formation, expansion and reorganization of their membranes and organelles in order to successfully multiply in their host. Application of lipid dyes revealed that the PVM is formed by an invagination process from the host cell plasma membrane^3^, and GFP-targeting to parasite organelles has greatly expanded our knowledge of how the apicoplast, mitochondria and nuclei are formed and organized during parasite development^4, 5^.

In contrast, we know very little about the biology of the PPM. A highly valuable insight into the structure and organization was first provided by electron microscopy analysis^6–12^. More recently, the merozoite surface protein 1 (MSP1) was shown to be expressed in the PPM of late liver stage parasites, which allowed the study of PPM invagination by immunofluorescence analysis (IFA)^13^. Further insight into PPM formation of this stage was obtained in the same study by the expression of a red fluorescent protein in the parasite cytosol that enabled the indirect visualization of PPM invagination by live-cell microscopy^13^. GFP-tagging of the PPM-localized cation-transporting P-type ATPase (PfATP4) allowed direct visualization of the PPM in living blood stage parasites^14^. This was key to understanding the mechanism of action of imidazopyrazines, a new antimalarial compound class that influence PPM development by blocking a lipid kinase involved in this process^15^. Furthermore, GFP-tagging of the PPM-localized putative sphingomyelin-synthethase (PfSMS1) allowed studying the interaction of the PPM with the inner membrane complex during blood stage development^16^. However, a similar PPM reporter parasite line for the mosquito and liver stages of the parasite has not so far been established.

In this study, we describe the generation of a reporter parasite line by GFP-tagging of a non-essential PPM-localized protein and follow plasma membrane development in living parasites through the entire *Plasmodium* life cycle. By combining the PPM reporter with a reporter for the parasite endoplasmic reticulum (ER), we show that the ER forms contact sites with the PPM that might be important for non-vesicular lipid transport between both membrane systems.

## Results

### Identification of a *Plasmodium* plasma membrane reporter

We recently identified *Plasmodium berghei* phospholipase (PBANKA_112810) as a liver stage-expressed protein that is involved in parasite egress from host hepatocytes^17^. In the same database search, we identified the so far uncharacterized PPM resident protein PBANKA_080940, henceforth referred to as *P. berghei* Plasma Membrane Protein 1 (PbPMP1). PbPMP1 is a 404 amino acids protein, which is conserved among *Plasmodium* species and contains a predicted phospholipase C/P1-S1 nuclease domain in addition to a signal peptide and a C-terminal transmembrane domain (plasmoDB.org). An RNAseq study, primarily of blood stage *P. berghei* parasites, showed highest expression of PbPMP1 in schizont stages, in comparison to rings, trophozoites, gametocytes and ookinetes^18^. Furthermore, PbPMP1-derived peptides were detected in a study comparing the proteome of male and female gametocytes, indicating expression at this stage^19^.

**Figure 1:**
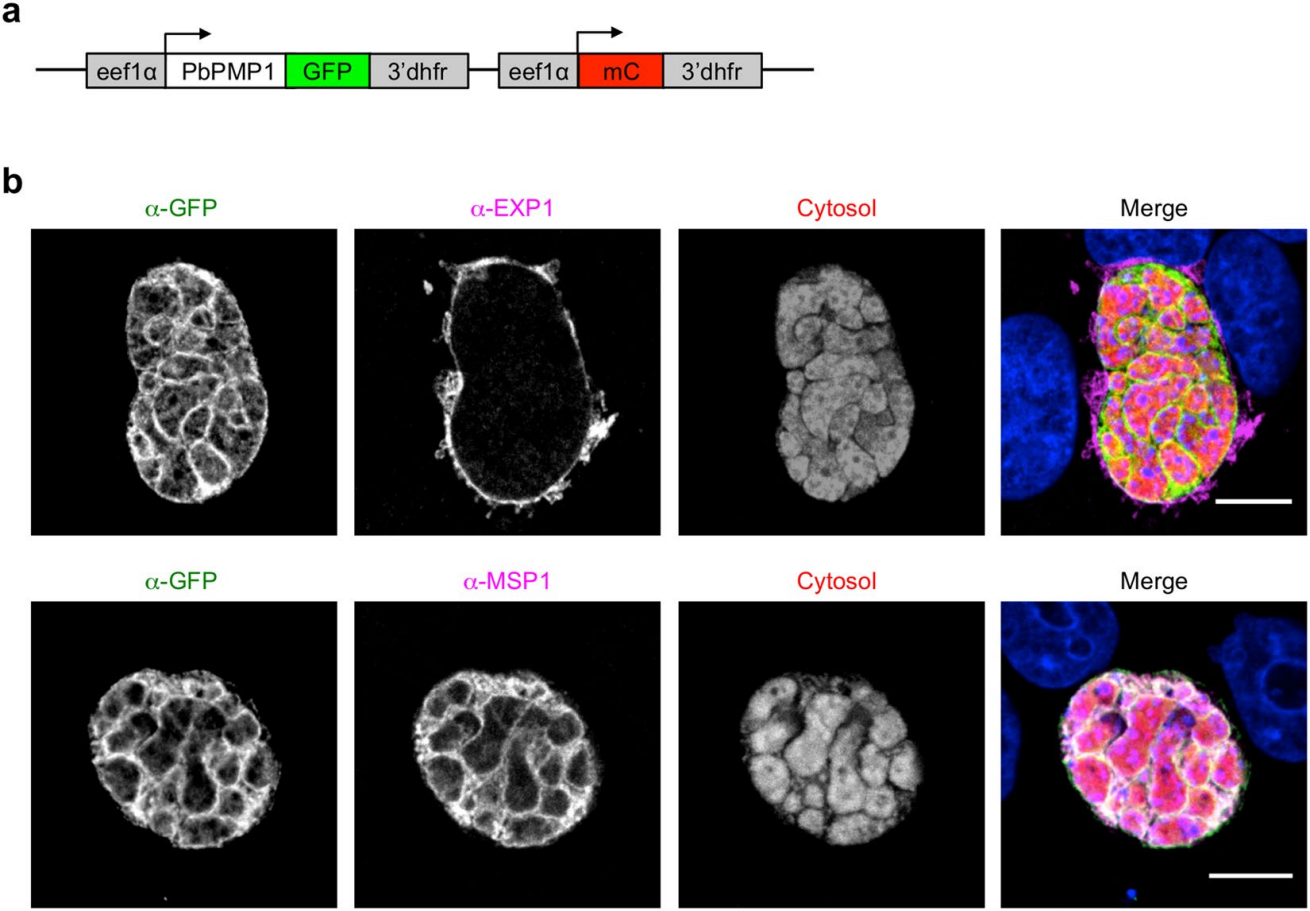
Generation and immunofluorescence analysis of PbPMP1-GFP parasites. (**a**) Schematic representation of the pL0017^C^PbPMP1-GFP-^C^mCherry plasmid. The PbPMP1-GFP fusion protein and a cytosolic mCherry were both expressed under the control of the constitutive *eef1α* promoter. The 3′-UTR was taken from Pbdhfr/ts. (**b**) PbPMP1-GFP localizes to the PPM in liver stage parasites. HeLa cells were infected with PbPMP1-GFP parasites and fixed at 54 hours post-infection (hpi). IFA was performed using antisera against the PPM marker MSP1 and the PVM marker EXP1. The PbPMP1-GFP signal was enhanced by staining with an anti-GFP antiserum (green). MSP1/EXP1 (purple). Parasite cytosol (red). The merged channels additionally contain DAPI-stained nuclei (blue). Scale bars = 10 µm.

To determine PbPMP1 localization, we generated the plasmid pL0017^C^PbPMP1-GFP^C^mCherry, encoding a PbPMP1-GFP fusion protein in addition to cytosolic mCherry, both expressed under the control of the constitutive *P. berghei eef1α* promoter (Fig. 1a). This vector integrates by single crossover recombination into either the *c-* or *d-ssu-rRNA* locus of *P. berghei* and conveys resistance to pyrimethamine. We transfected it into blood stages schizonts and obtained transgenic PbPMP1-GFP parasites after drug selection. Successful integration into the *c-ssu-rRNA* locus was confirmed_ by PCR (Supplementary Fig. S1a,b). To investigate PbPMP1-GFP parasite liver stage development, mosquitoes were infected by blood feeding on mice harboring PbPMP1-GFP parasites and resulting sporozoites were isolated from salivary glands. We used the isolated sporozoites to infect HeLa cells, which have been proven to be highly susceptible to *P. berghei* infection and to support successful development of liver stage parasites^20^. We first investigated fully developed cytomere stage parasites by confocal imaging of fixed and stained infected cells. This stage is particularly suited for localization studies, since parasites are very large at this time point and the PPM already started to invaginate, allowing a clear differentiation between the PVM and the PPM. IFA revealed that PbPMP1-GFP co-localized with the PPM marker MSP1 but not with exported protein 1 (EXP1), which was used to visualize the PVM (Fig. 1b). Importantly, expression of PbPMP1-GFP did not influence the development of parasites, as PbPMP1-GFP parasites showed normal numbers of sporozoites in the salivary glands (Supplementary Fig. S1c) and progressed normally through liver stage development (Supplementary Fig. S1d,e).

To also analyze the function of endogenous PbPMP1, we generated PbPMP1-knockout parasites by a targeted deletion of the PbPMP1 coding sequence by double crossover homologous recombination (Supplementary Fig. S2a,b). However, we did not observe any differences to wild-type parasites throughout the life cycle (Supplementary Fig. S2c–i), indicating that endogenous PbPMP1 is dispensable for parasite development.

### PbPMP1-GFP localizes to the PPM in live parasites throughout the *Plasmodium* life cycle

Having established PbPMP1-GFP as plasma membrane marker protein, we next sought to investigate PbPMP1-GFP localization and thereby plasma membrane organization by live-cell imaging throughout the *Plasmodium* life cycle. We first examined PbPMP1-GFP blood stage parasites. Here, although rather weak, the GFP signal was found to surround the cytoplasmic mCherry signal in trophozoites, merozoites and gametocytes, suggesting that PbPMP1-GFP also localizes to the PPM during blood stage development (Supplementary Fig. S3a–c). This finding was further confirmed by IFA of blood stage schizonts, where PbPMP1 was co-localizing with the plasma membrane marker MSP1 (Supplementary Fig. S4). PbPMP1 was also localized at the PPM of ookinetes, in which PbPMP1-GFP was additionally located in intracellular dots, possibly representing newly synthesized PbPMP1 during transport to the plasma membrane (Supplementary Fig. S3d).

**Figure 2:**
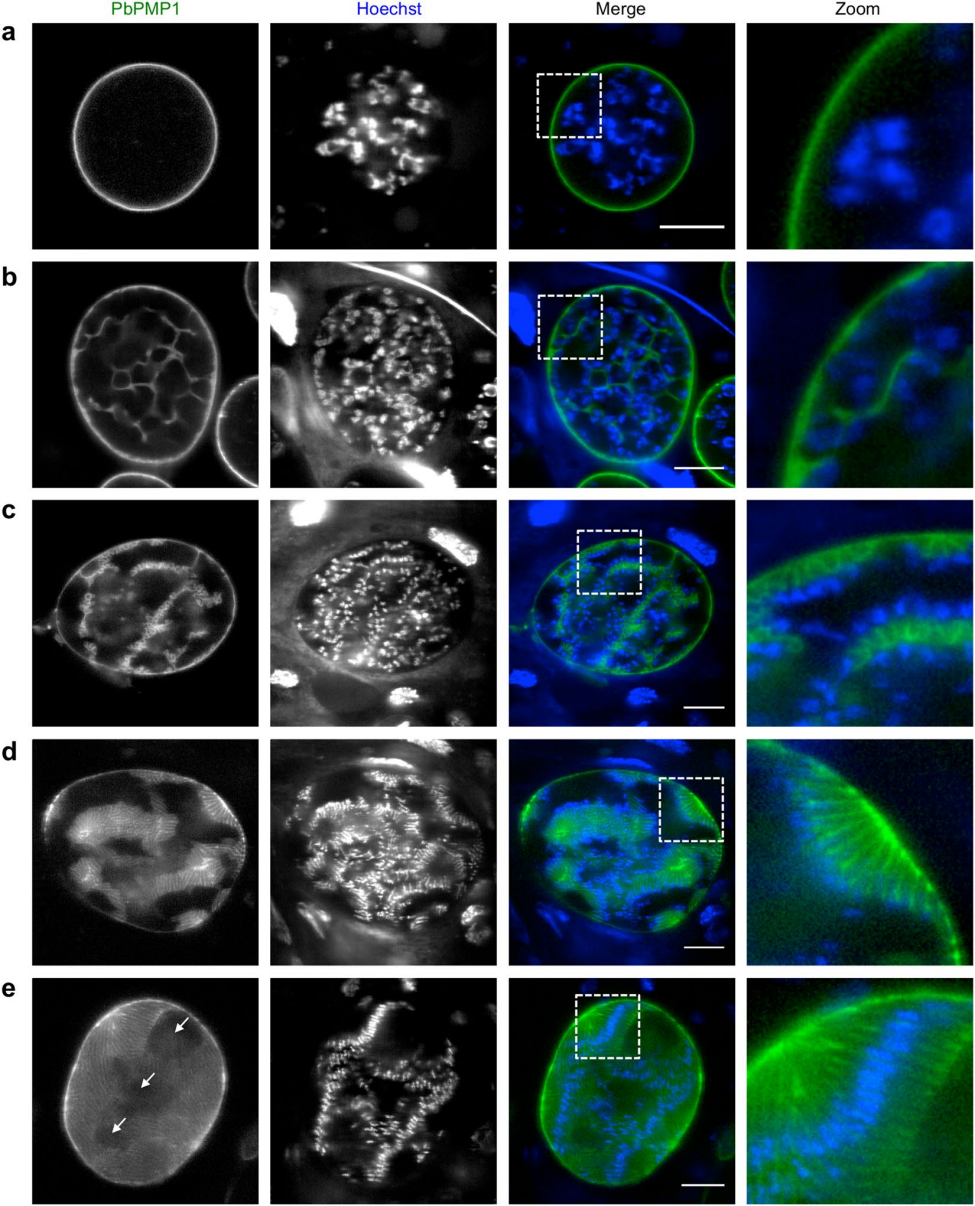
Visualization of the oocyst plasma membrane in live PbPMP1-GFP parasites. Midguts of PbPMP1-GFP parasite-infected mosquitoes were isolated at day 7, 9 and 11 after the infectious blood meal and were analyzed by confocal microscopy. **(a)** Oocyst at day 7 without PPM invaginations. (**b**) and (**c**) Oocysts at day 9 and 11, in which the PPM has started to form invaginations. Note that the previously randomly localized nuclei start to align along the newly formed membranes and that the PPM undergoes further invaginations close to these nuclei in (**c**). (**d**) and (**e**) Sporozoite formation in oocysts at day 11. Note the residual bodies; regions of cytoplasm not incorporated into forming sporozoites (indicated with arrows). PbPMP1-GFP (green). DNA was stained with Hoechst 33342 (blue). Scale bars = 10 µm. For confocal z-stacks see also Supplementary Movies S1 and S2.

Subsequently, we analyzed the development of PbPMP1-GFP oocysts at 7, 9 and 11 days post mosquito infection. Previous electron microscopy studies on the development of sporozoites within oocysts already provided insight on how membrane formation occurs during sporozoite morphogenesis^6–8, 10–12^. The PPM was shown to retract from the oocyst wall and by continuous fusion of vesicles to invaginate into the oocyst, ultimately resulting in the formation of cytoplasmic islands called sporoblasts, from which sporozoites were formed from the periphery by a budding process. We observed a strong GFP signal in the periphery of all oocysts without any signs of invagination at day 7 post-infection, indicating that PbPMP1-GFP also labels the PPM in this parasite stage (Fig. 2a). At this time of development, the nuclei had already started dividing but seemed to be randomly distributed throughout the oocyst. In line with the electron microscopy studies, in later stages of oocyst development at 9 and 11 days after the infectious blood meal, PbPMP1-GFP started to form a seemingly interconnected network within oocysts. The nuclei were thereby found to align along the newly formed membranes, at sites where subsequent sporozoite formation was initiated (Fig. 2b–d, for a confocal z-stack see Supplementary Movie S1). Finally, PbPMP1-GFP was surrounding individual sporozoites within oocysts, which still contained portions of cytoplasmic material that had not been incorporated into forming sporozoites, these presumably being the formerly described residual bodies^6^ (Fig. 2e, for a confocal z-stack see Supplementary Movie S2). Importantly, in the individual oocysts examined, sporozoites appeared to be all at the same stage of development, indicating that generation of sporozoites inside oocysts seems to be a fairly synchronous event rather than a continuous budding process. Biologically, synchronous development makes sense, as at the time of oocyst rupture all sporozoites are at the same developmental stage. In isolated salivary gland sporozoites and transforming sporozoites inside host HeLa cells, PbPMP1-GFP was similarly found at the parasite periphery, although the fluorescence was much weaker as compared to oocysts (Supplementary Fig. S5a,b). We therefore further investigated this localization by fixation of PbPMP1-GFP salivary gland sporozoites and staining them with antisera against GFP and the circumsporozoite protein (CSP). This revealed a partial co-localization between CSP and PbPMP1. Similar to what we observed in ookinetes (Fig. S3d), PbPMP1 was also found in intracellular structures within the parasite (Supplementary Fig. S6).

**Figure 3:**
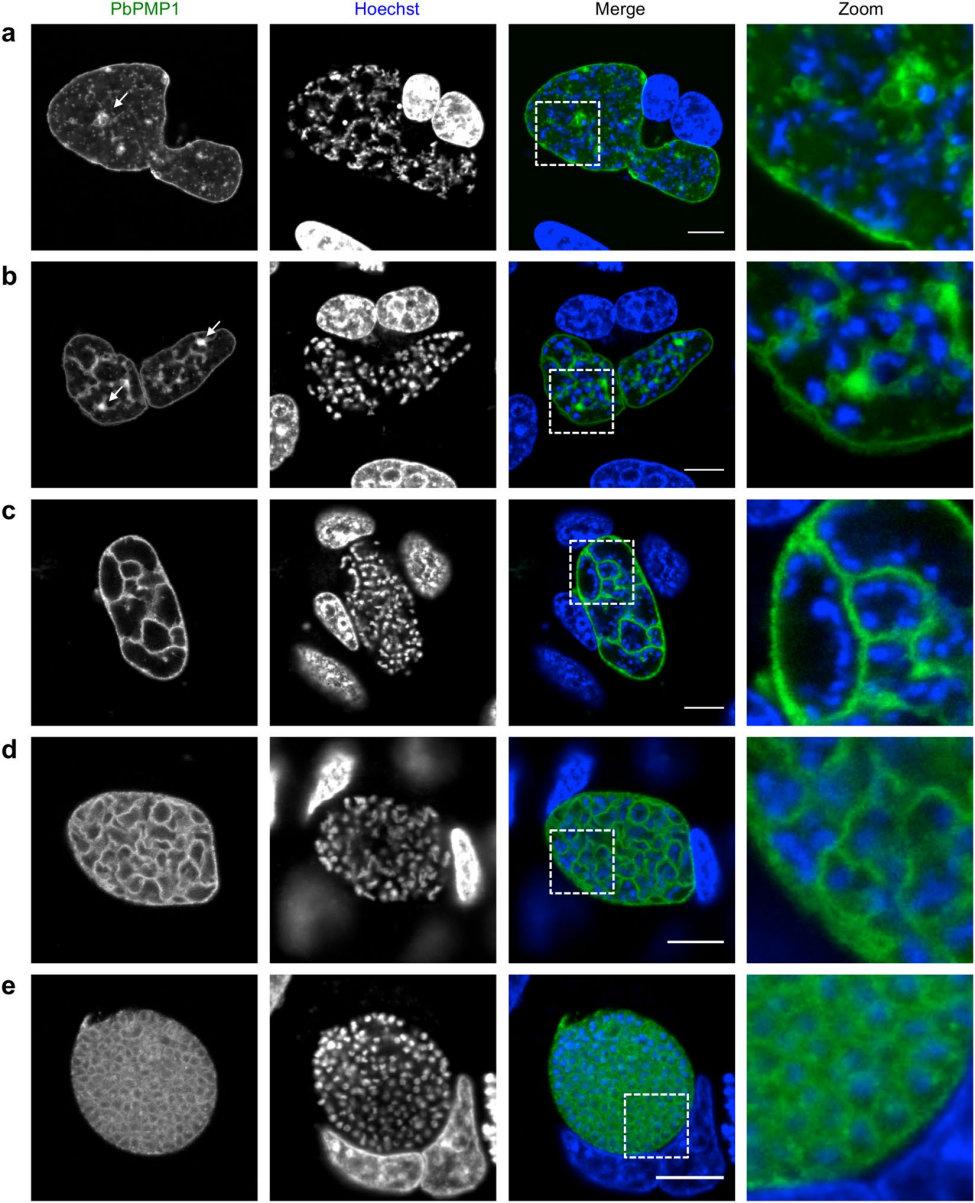
Plasma membrane morphology in live PbPMP1-GFP late liver stage parasites. HeLa cells were infected with PbPMP1-GFP parasites and analyzed by confocal microscopy at 48 hpi (**a**,**b**) and 54 hpi (**c**,**d**,**e**). PbPMP1-GFP (green). DNA was stained with Hoechst 33342 (blue). Note the appearance of membrane accumulations (indicated with arrows), from which new membranes frequently appeared to originate and connect to the surrounding PPM. Scale bars = 10 µm. For earlier liver stage parasites see also Supplementary Fig. S5.

In early schizonts during liver stage development, PbPMP1-GFP was found at the plasma membrane as well and appeared as intracellular small vesicles, which was comparable to its localization in ookinetes (Supplementary Fig. S5c,d). In very late schizont stages at around 48 hpi, a different kind of larger PbPMP1-GFP-positive structures could be observed within parasites (Fig. 3a). These were later often interconnected and seemed to form part of a membranous network (Fig. 3b). At 54 hpi, in line with our IFA analysis (Fig. 1b), PbPMP1-GFP localized to the invaginated membrane of cytomere stages and the previously randomly distributed nuclei aligned in close proximity to the invaginated PPM (Fig. 3c,d). Finally, the PbPMP1 signal was found to surround individual merozoites (Fig. 3e).

In conclusion, these findings show that PbPMP1-GFP visualizes the PPM through the entire *Plasmodium* life cycle and serves as an excellent marker for this membrane especially during oocyst and liver stage development.

### Time-lapse imaging of PbPMP1-GFP parasites reveals synchronous merozoite formation

**Figure 4:**
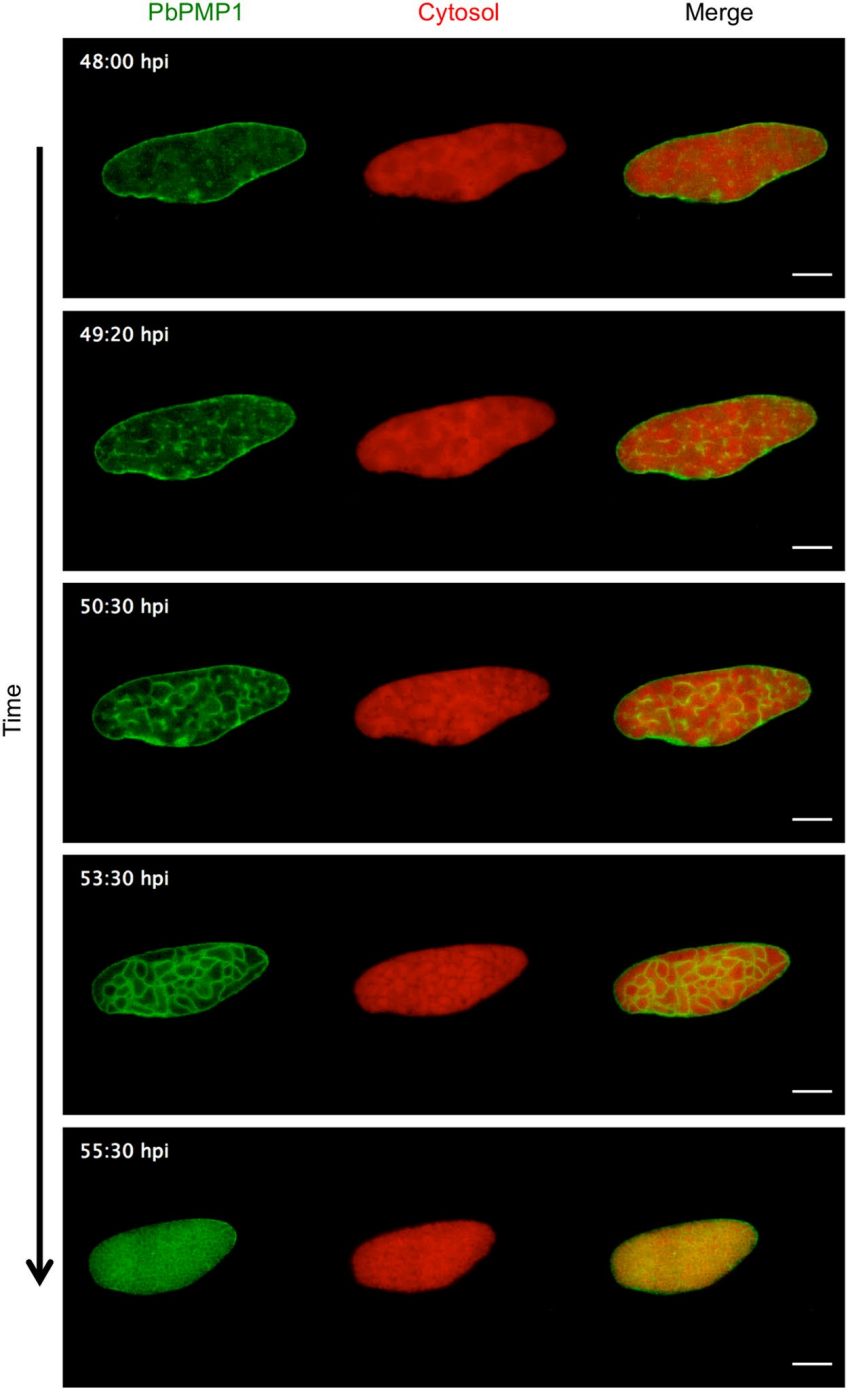
Visualization of PPM dynamics during late liver stage development by live-cell time-lapse microscopy. Stills from a representative movie of PbPMP1-GFP late liver stage development. HeLa cells were infected with PbPMP1-GFP parasites and confocal live-cell imaging was started at 48 hpi. PbPMP1-GFP (green). Parasite cytosol (red). Time points of imaging are indicated. Scale bars = 10 µm. See also Supplementary Movie S3.

In order to further understand the dynamics of PPM formation during liver stage development, we analyzed PbPMP1-GFP expressing liver stage parasites by live-cell time-lapse microscopy. At 48 hpi, some small vesicular structures were present within the parasite and with time these vesicles appeared to coalesce and formed larger membrane accumulations (Fig. 4 and Supplementary Movie S3). New membranes were frequently observed to originate from these accumulations. This was followed by a fusion of the newly formed membranes to other membranes and further branching, whereby the membranous network typical of the cytomere stage was generated at around 54 hpi. Thereafter, the parasite cytoplasm was further subdivided by repeated synchronous invagination events of the PPM until individual merozoites were formed (Fig. 4 and Supplementary Movie S3).

Previous studies analyzed the process of hepatic merozoite formation by electron microscopy^9, 11^. Here, a cleft formation by fusion of peripheral vacuoles was described, which divided the cytoplasm of liver stage parasites into a labyrinth-like structure. It was further proposed that this leads to the formation of cytoplasmic islands known as meroblasts, from which merozoites were continuously produced by a budding process at the periphery. Our live-cell imaging experiments following the process of hepatic merozoite formation over time confirm the proposed invagination process, but suggest that merozoite formation occurs rather by repeated synchronous invagination events of the PPM, which subdivide the parasite cytoplasm until merozoites have been formed.

### Photobleaching experiments using PbPMP1-GFP parasites confirm PPM invaginations

Although it is generally accepted that daughter cells are generated by invagination processes of the PPM, it is not fully understood how the initial membranes are formed that first subdivide the parasite cytoplasm before further membrane invaginations occur. One possibility would be that initial membranes are formed by expansion of internal membranes and fusion of these membranes to the surrounding PPM, a scenario that has been suggested to occur during sporozoite formation in *Plasmodium falciparum* oocysts, where expansion of the cisternal space of the ER was reported to be responsible for subdivision of the oocyst cytoplasm^8^. Alternatively, initial membranes could form from the existing PPM by invaginations, which might be supported by fusion of peripheral vacuoles with the PPM, as suggested for example by electron microscopy analysis of *P. berghei* liver stage parasites^9^.

When we followed PPM formation in liver stage parasites over time, we observed the coalescence of smaller membrane vesicles to form larger membrane accumulations. Following this, membranes were frequently observed to grow out of these membrane accumulations and to connect to each other (Figs. 3a,b and 4). To investigate the origin of these membrane accumulations in detail, we next performed Fluorescence Loss In Photobleaching (FLIP) experiments with PbPMP1-GFP liver stage parasites. FLIP is a powerful live-cell imaging-based tool that has often been used to investigate whether two structures are interconnected or to demonstrate the continuity of intracellular organelles^21–23^. In a FLIP experiment, a cell expressing fluorescently labeled proteins is repeatedly bleached within a small region, while images of the whole cell are taken with reduced laser power between the bleaches. Any regions of the cell that are connected to the area being bleached will gradually lose fluorescence due to lateral movement of mobile proteins into this area, whereas the fluorescence in unconnected regions will not be affected^24^.

**Figure 5:**
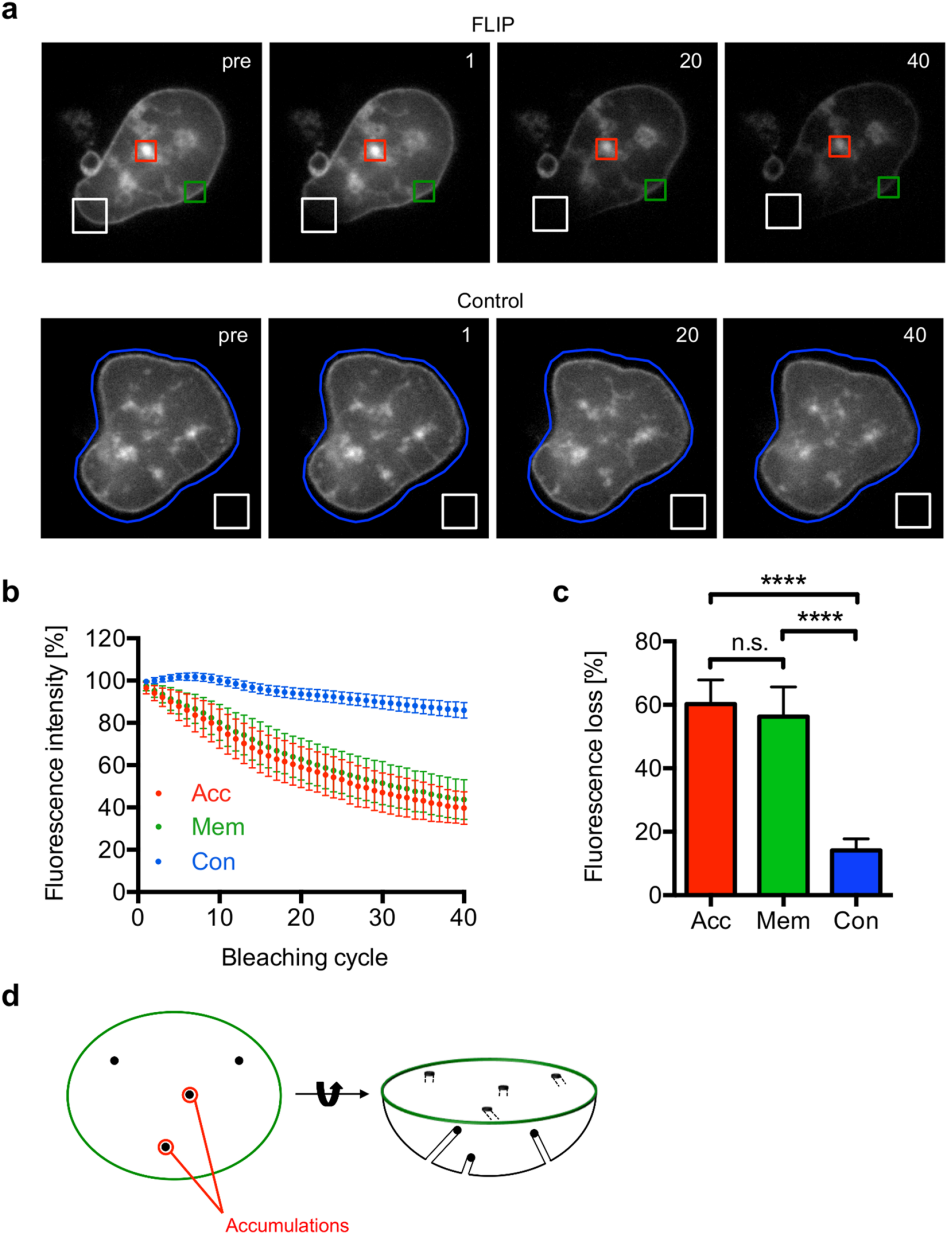
FLIP experiments show that early membrane accumulations are connected to the surrounding PPM. HeLa cells were infected with PbPMP1-GFP parasites and a possible connection of membrane accumulations to the surrounding PPM was analyzed by FLIP experiments at 48 hpi. In each FLIP experiment, a single region of the surrounding PPM (white square) was repeatedly bleached and the fluorescence intensity of a membrane accumulation (Acc, red square) and another region on the surrounding plasma membrane (Mem, green square) was determined. As a control (Con), bleaching was performed outside of the parasite (white square) and the fluorescence intensity of the whole parasite was measured (blue circle). For all experiments, an image was acquired before bleaching and the corresponding fluorescence intensity was set to 100%. (**a**) Representative stills from a FLIP and a control parasite, with bleaching cycles indicated in the upper right. Pre = prebleaching. (**b**) Fluorescence intensity over time. (**c**) Statistical evaluation of the loss of fluorescence after 40 bleaching cycles. Shown are means with SD of 21 FLIP and 21 control parasites, which were obtained in two independent experiments. For statistical analysis, a one-way ANOVA followed by a Holm-Sidak multiple comparison test was performed (**** p < 0.0001, n.s. = not significant). (**d**) Schematic drawing of first membrane invaginations that presumably appear as membrane accumulations in a two-dimensional representation.

To analyze the connection of the initial membrane accumulations to the PPM, we repeatedly bleached a small area of the surrounding PPM and determined the loss of fluorescence intensity, firstly on a region encompassing a single membrane accumulation and, secondly, on another region located at the surrounding PPM. In contrast to unbleached control parasites, PPM bleaching resulted in a significant and similar loss of fluorescence intensity of both measured regions (Fig. 5a–c) indicating that initial membrane accumulations are connected to the surrounding plasma membrane of the parasite. This, in turn, suggests that the membrane accumulations in the parasite represent invaginations from the surrounding PPM (Fig. 5d). These data, however, do not exclude the possibility that membrane accumulations also could have formed from internal membranes, which later fused to the PPM.

### Double-fluorescent parasites reveal interactions of the PPM with the parasite ER

**Figure 6:**
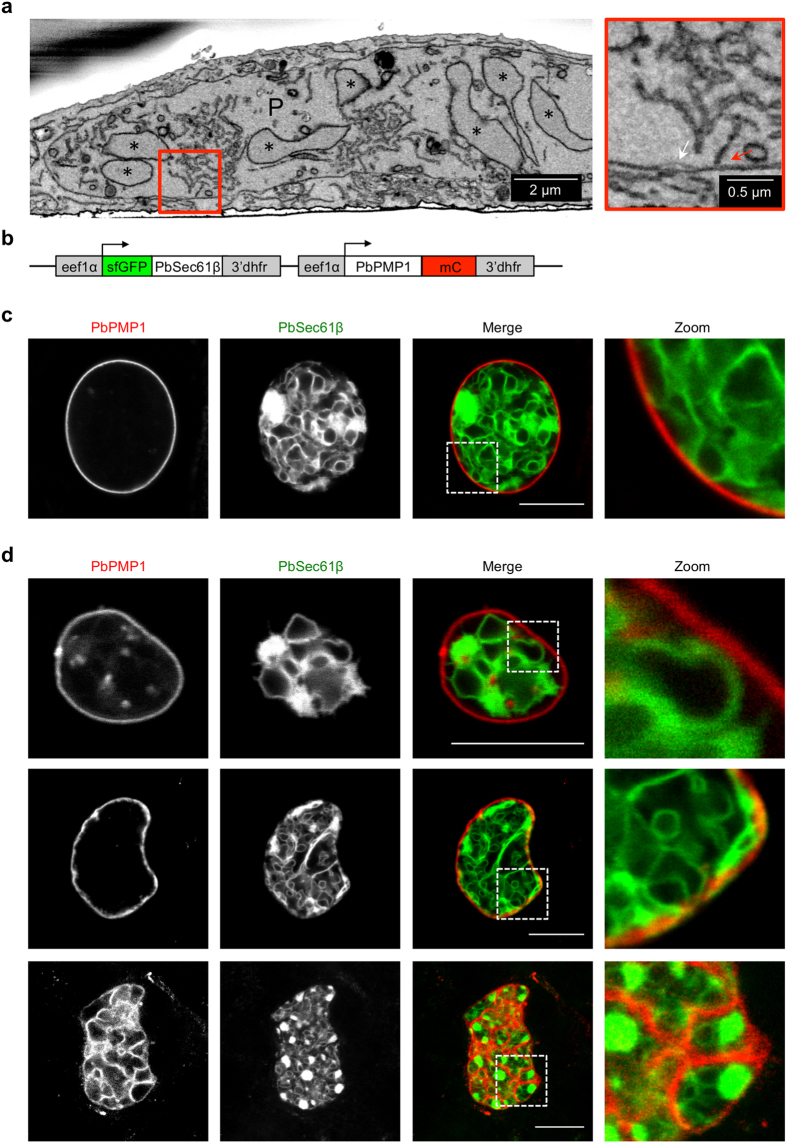
Double-fluorescent parasites reveal interactions of the PPM with the parasite ER during oocyst and liver stage development. (**a**) SBFSEM of the *P. berghei* ER. HeLa cells infected with mCherry-expressing parasites were fixed at 48 hpi and osmium-stained for EM. Cells were vertically cut and images were taken by SBFSEM. The boxed area is shown at a higher magnification on the right side and shows an ER extension (red arrow) to the parasite PM and or PVM (white arrow). P, parasite; asterisks, parasite nuclei. (**b**) Schematic representation of the pL0017^C^sfGFP-PbSec61β-^C^PbPMP1-mCherry plasmid. The sfGFP and mCherry fusion proteins were both expressed under the control of the constitutive *eef1α* promoter. The 3′-UTR was taken from Pbdhfr/ts. (**c**) and (**d**) Interactions of the parasite ER and the PPM in oocysts and liver stage parasites. (**c**) Midguts of sfGFP-PbSec61β/PbPMP1-mCherry parasite-infected mosquitoes were isolated at day 7 after the infectious blood meal and were analyzed live by confocal microscopy. (**d**) HeLa cells were infected with sfGFP-PbSec61β/PbPMP1-mCherry parasites and analyzed live by confocal microscopy at 24 hpi (upper row) and 48 hpi (two lower rows). SfGFP-PbSec61β (green), PbPMP1-mCherry (red). Extensions of the ER in contact with the surrounding PPM were found in all oocysts and liver stages examined (a total of 20 oocysts analyzed at day 7 and 9 post-feed, a total of 60 liver stages at 24 hpi and a total of 60 liver stages at 48 hpi assessed). For confocal z-stacks see also Supplementary Movies S4 and S5. For a time-lapse movie of sfGFP-PbSec 61β/PbPMP1-mCherry parasite liver stage development see also Supplementary Movie S6. Scale bars correspond to 10 µm, if not labelled differently.

We recently characterized the morphology of the ER in *P. berghei* by GFP-tagging of the ER marker PbSec61β^20^. The parasite ER forms an interconnected network throughout the parasite, with perinuclear and peripheral localizations, in addition to large accumulations. Interestingly, extensions of the ER appear to form contact sites with the PPM or the PVM, which are visible by serial block face scanning electron microscopy (SBFSEM, Fig. 6a and ref. 20).

**Figure 7:**
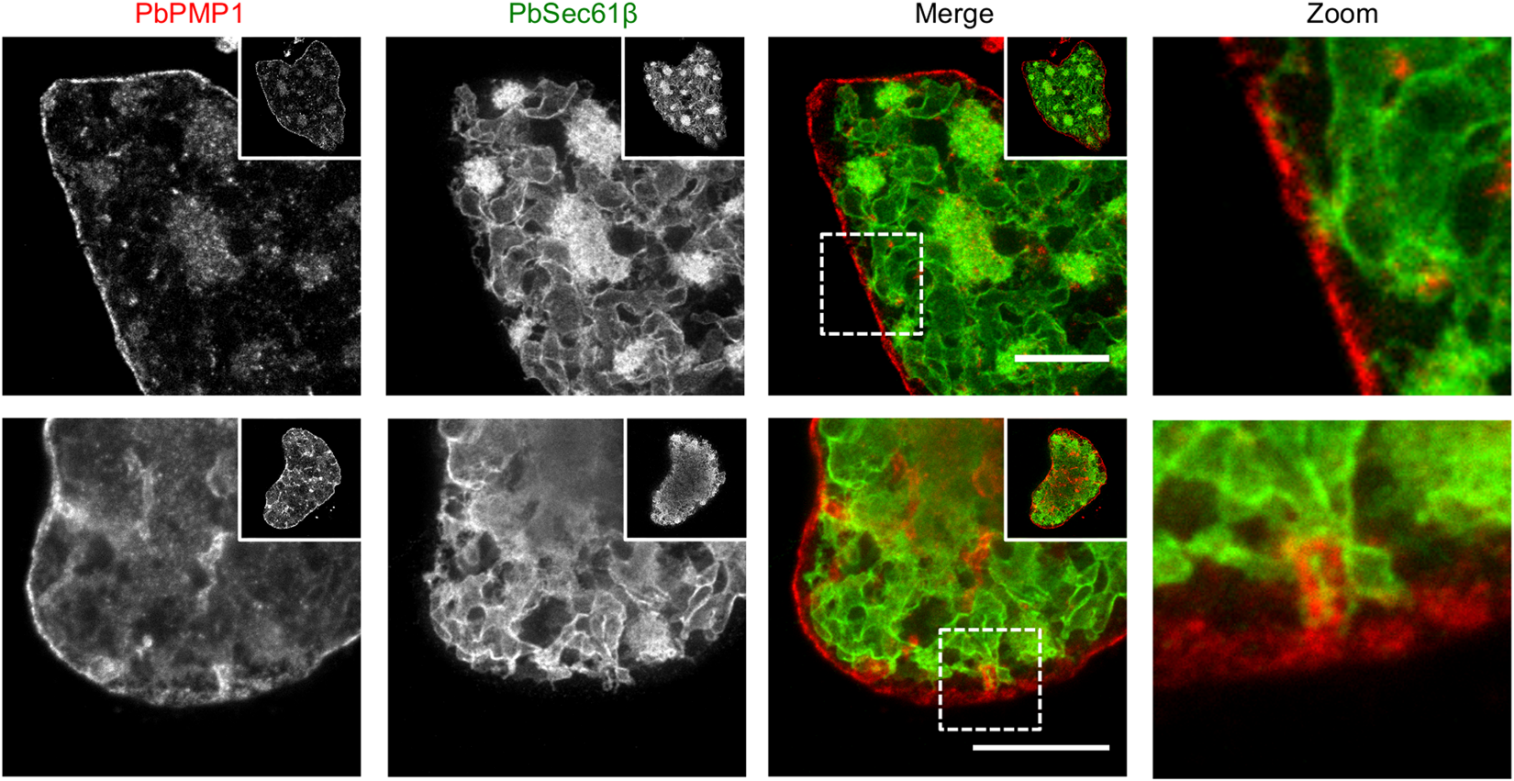
Super-resolution STED microscopy of double-fluorescent sfGFP-PbSec61β/PbPMP1-mCherry liver stage parasites. HeLa cells were infected with sfGFP-PbSec61β/PbPMP1-mCherry parasites and fixed at 48 hpi. The sfGFP-PbSec61β (green) and the PbPMP1-mCherry (red) signals were enhanced by staining with specific antisera and parasites were analyzed by STED super-resolution microscopy. Scale bars = 10 µm.

To investigate these interactions in further detail, we aimed to visualize the parasite ER in combination with the PPM in living parasites. We thus cloned a construct that leads to expression of a superfolder GFP (sfGFP)-PbSec61β and a PbPMP1-mCherry fusion protein both under control of the constitutive *eef1α* promoter (Fig. 6b). After transfection of this plasmid into blood stage schizonts and confirmation of successful integration into the *d-ssu-rRNA* locus by PCR (Supplementary Fig. S1b), we analyzed the interaction of the PPM with the ER during oocyst and liver stage development by live-cell confocal microscopy. Remarkably, in all oocysts and liver stages examined, extensions of the ER were found in contact with the surrounding PPM (Fig. 6c,d and Supplementary Movies S4–S6). We further characterized these contacts by imaging double-fluorescent liver stage parasites using stimulated emission depletion (STED) microscopy, which typically gives a resolution of 30–80 nm^25^. This revealed that the PPM seemingly interlaces with the ER at these contact sites (Fig. 7), suggesting a close interaction between both membrane systems during parasite development.

## Discussion

**Figure 8:**
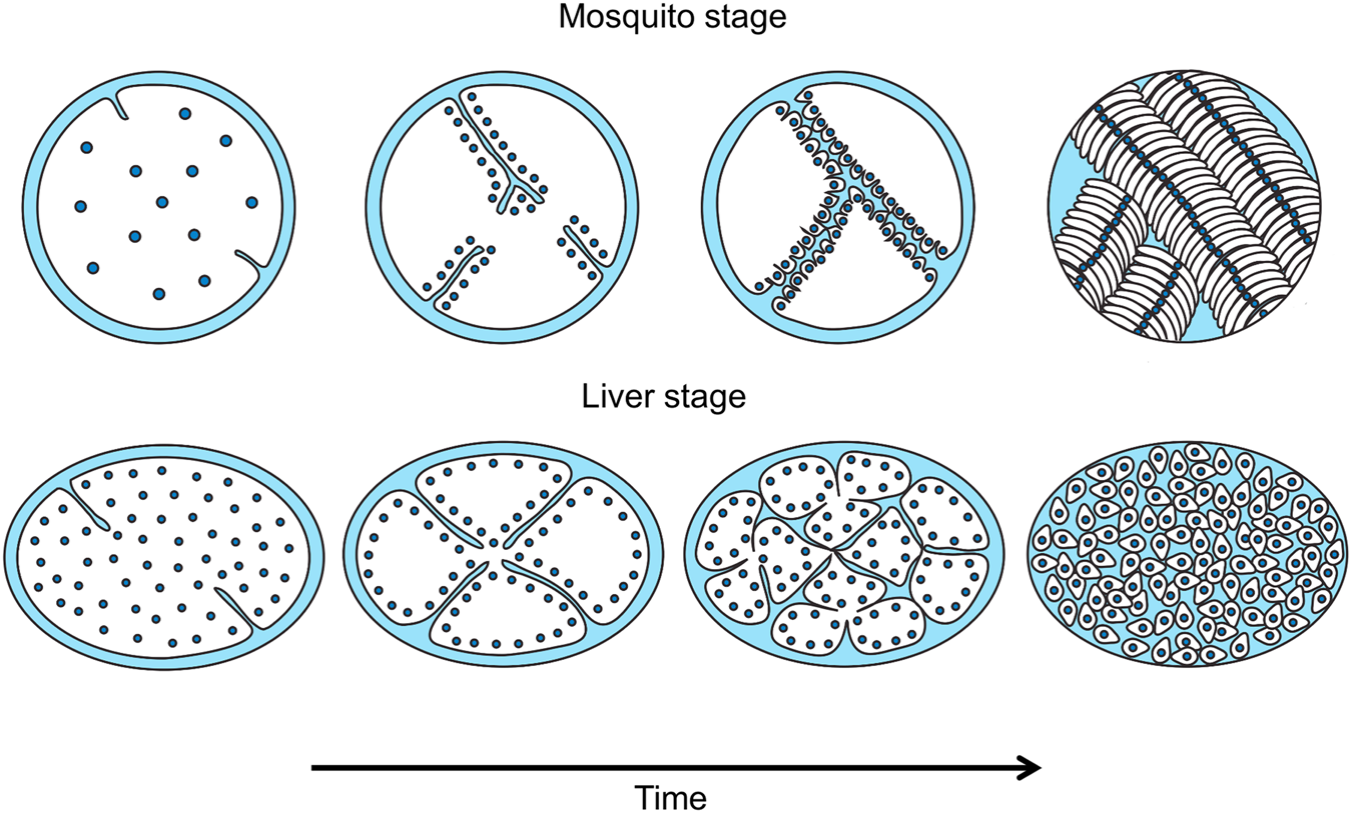
Model of PPM development during formation of sporozoites and hepatic merozoites. The PPM is shown in black and the nuclei in blue. When nuclear division is about to complete, the PPM starts to form invaginations, which further expand into the cytoplasm. The newly formed membranes fuse to each other and undergo additional branching and nuclei align along these membranes. In a synchronous manner, membranes subsequently form around the individual nuclei to generate daughter parasites, involving further repeated invagination events in the case of hepatic merozoites.

The generation of *P. berghei* parasites in which PbPMP1-GFP localizes to the PPM has allowed us to study PPM formation at every stage of the life cycle in living parasites. Previous electron microscopy studies have already provided a very solid basis to our understanding of PPM organization and suggested a continuous peripheral budding of sporozoites from the sporoblast and hepatic merozoites from the meroblast^6–12^. Our own results obtained by live-cell imaging extend these findings, in that they show that sporozoites and merozoites are produced in a rather synchronous process. In addition to this, our findings highlight the fact that PPM formation during sporozoite and hepatic merozoite morphogenesis basically follows the same mechanism, which we have summarized in Fig. 8: When nuclear division is close to completion, the surrounding PPM starts to form invaginations, which expand into the cytoplasm of oocysts or liver stage parasites. Membranes then fuse to each other and undergo additional branching, and nuclei align along the newly formed membranes. Membranes subsequently form in a synchronous manner around the individual nuclei to generate tens of thousands of infectious daughter parasites, involving further repeated invagination events in the case of hepatic merozoites.

Our time-lapse imaging data of PbPMP1-GFP liver stage parasites clearly illustrates how complex and carefully timed the process of PPM formation is. It will now be interesting to investigate how this is achieved on a molecular level. In this respect, PbPMP1-GFP parasites will be very useful as a PPM reporter parasite line. One of the key questions will be, for example, how the parasite controls the various membrane fusion events during daughter cell formation. Possible candidates would be the SNARE (soluble N-ethylmaleimide-sensitive factor attachment protein receptors) proteins, which have been recognized as key components of protein complexes that drive membrane fusion in eukaryotic cells (reviewed in ref. 26). Interestingly, 18 SNARE proteins have been predicted for *P. falciparum* ^27^, out of which one protein was already shown to localize to the PPM of *P. falciparum* blood stages^28^. The detailed molecular function of SNARE proteins in *Plasmodium*, however, has so far not been investigated. Another interesting question will be, whether the parasite cytoskeleton is involved in the complex membrane reorganization processes during cytokinesis and if so, how the linkage between cytoskeleton and PPM is achieved. The two isotypes of α-tubulin have been shown to be essential for *Plasmodium* asexual blood stage development^29^ but a potential association of these to the plasma membrane, as previously described for other organisms (reviewed in ref. 30), has not been studied so far.

By combining the PPM reporter with a reporter for the parasite ER, we were able to characterize the interaction of both membrane systems by live-cell imaging and STED super-resolution microscopy. We thereby provided evidence for potential membrane contact sites (MCS) formed between the ER and the PPM during oocyst and liver stage development. This extends our previous findings, which showed that extensions of the parasite ER are in close association with the PVM marker protein EXP1 in liver stage parasites^20^. The interaction of the parasite ER and the PPM might be of importance for lipid transport processes within the parasite, as lipid transport is greatly facilitated at MCS. These are defined as small cytosolic gaps (10–30 nm) between two membranes that enable the transport of Ca^2+^, lipids and metabolites by a non-vesicular transport mechanism. The transport of lipids is thereby often assisted by specific lipid-transfer proteins. MCS are established and maintained in a transient or durable fashion by tethering structures, which keep the two membranes in close proximity, although fusion between the membranes does not occur (reviewed in ref. 31).

In *Plasmodium*, most lipids seem to be scavenged from the host, but by using a functional FASII system in the apicoplast, parasites are also able to synthesize lipids *de novo*. Interestingly, depletion of proteins involved in the FASII system did not affect blood and mosquito stage development but completely inhibited late liver stage development in *Plasmodium yoelii*, indicating that *Plasmodium* parasites depend on *de novo* fatty acid synthesis only for this stage (reviewed in ref. 32). It is likely that fatty acids synthesized in the apicoplast are ultimately incorporated into membrane phospholipids necessary for daughter cell formation. Recently, research in *P. yoelii* liver stages revealed that enzymes for the synthesis of the phospholipid precursor phosphatidic acid are targeted to the apicoplast and the ER^33^. The authors proposed a model in which apicoplast-derived lysophosphatidic acid is converted into phosphatidic acid at the ER and then metabolized into phospholipids. However, how the ER-synthesized lipids would then be distributed to organelles and to the PPM is not understood. The findings of the present study suggest potential MCS between ER and PPM and thereby provide a possible explanation of how phospholipid transfer might occur. In this respect, it is interesting to note that studies in the related apicomplexan parasite *Toxoplasma gondii* also revealed the existence of MCS between the apicoplast and the ER and it has been suggested that these sites might allow for the subcellular distribution of phospholipids in the parasite^34^. Clearly, further research is required to obtain a more mechanistic view on the role of MCS for lipid transport processes in *Plasmodium* parasites, for which the underlying molecular machinery will have to be identified and characterized in the future.

In conclusion, this study identified PbPMP1-GFP parasites as a powerful tool to investigate the fascinating process of PPM organization during *Plasmodium* development by live-cell microscopy. Furthermore, it provided evidence for the formation of contact sites between the ER and the PPM during oocyst and liver stage development. In the future, PbPMP1-GFP parasites will be useful to screen for drugs that interfere with PPM development and to study transport processes to and beyond the PPM by live-cell imaging. Additionally, combining PbPMP1 with markers for other parasite membranes, such as the PVM or the inner membrane complex, will help to further study the dynamic interaction of membrane systems during parasite development, which will altogether greatly expand our knowledge of membrane biology in the *Plasmodium* parasite.

## Methods

### Ethics statement

All experiments performed at the University of Bern were conducted in strict accordance with the guidelines of the Swiss Tierschutzgesetz (TSchG; Animal Rights Laws) and approved by the ethical committee of the University of Bern (Permit Number: BE109/13).

### Experimental animals

Mice used in the experiments were between 6 and 10 weeks of age and were from Harlan Laboratories or bred in the central animal facility of the University of Bern. Mosquito feeds were performed on mice anaesthetized with Ketavet/Domitor and all efforts were made to minimize suffering. The *in vivo* phenotype of PbPMP1-KO parasites was analyzed in female C57BL/6 mice, while for all other experiments male or female Balb/c mice were used.

### Mosquito infection

Infections of mice were initiated by intraperitoneal injection of *P. berghei* blood stabilates. When these mice had a parasitemia of 4%, 150 µl or 40 µl of infected blood were injected intraperitoneally or intravenously, respectively, into mice that had received an intraperitoneal injection of 200 µl phenylhydrazine (6 mg/ml in PBS) two to three days before. At day 3 or 4 after infection, mice with a parasitemia of at least 7% were anaesthetized for one hour to allow feeding of 150 female *Anopheles stephensi* mosquitoes. Mosquitoes were kept at 20.5 °C with 80% humidity and for infection experiments, sporozoites were isolated from infected salivary glands 16–27 days after the infective blood meal.

### Culture and infection of HeLa cells

HeLa cells (gift by Robert Menard, Pasteur Institute, Paris) were authenticated by STR DNA profiling (Microsynth) and cultured as described before^20^. HeLa cells were seeded onto coverslips or into live-cell imaging dishes and infected with *P. berghei* sporozoites as previously described^17^.

### Generation of PbPMP1-GFP and sfGFP-PbSec61β/PbPMP1-mCh parasites

The PbPMP1-GFP expression vector pL0017^C^PbPMP1-GFP^C^mCherry was generated by first amplifying the PbPMP1 coding sequence from blood stage cDNA using primer pair PbPMP1-GFP-fw/PbPMP1-GFP-rev and this was subsequently cloned into pL0017^35^ in frame with GFP using *BamHI* restriction sites. Subsequently, a constitutive mCherry expression cassette, which had been amplified before from the p^C^mCherry plasmid^36^ using primers eef1a-fw and 3dhfr-rev, was integrated using *KpnI* restriction sites.

The sfGFP-PbSec61β/PbPMP1-mCherry expression vector pL0017^C^sfGFP-PbSec61β ^C^PbPMP1-mCherry was generated by cloning the PbPMP1 coding sequence into the p^C^mCherry plasmid^36^ in frame with mCherry using *BamHI* restriction sites. Subsequently, the whole constitutive PbPMP1-mCherry expression cassette was amplified using primers eef1a-fw and 3dhfr-rev and cloned into the plasmid pL0017^C^sfGFP-PbSec61β^20^ using *KpnI* restriction sites.

Final plasmids were linearized by digestion with *ApaI* and *SacII* and transfected into blood stage schizonts of *P. berghei* ANKA parasites using standard methods of transfection^37^. Transfected parasites were selected by pyrimethamine (Sigma) in the drinking water of infected mice. Parasite genomic DNA was isolated from 0.05% saponin-treated infected red blood cells using the Nucleospin Blood QuickPure kit (Macherey-Nagel) and successful integration of the plasmid into the genome was confirmed by diagnostic PCR using GoTaq Flexi DNA polymerase (Promega). All primer sequences are listed in Supplementary Table S1. Sporozoite and liver stage development of PbPMP1-GFP parasites was compared to control parasites that constitutively express cytosolic mCherry either under the constitutive *P. berghei hsp70* or the *P. berghei eef1α* promoter^17, 36^.

### Parasite size measurement and detached cell analysis

5 × 10^4^ HeLa cells per well were seeded in 24-well plates and infected the next day with sporozoites. 48 hpi, parasite size (area) was determined by density slicing using ImageJ and infected cells were counted. At 65 hpi, the number of detached cells in the supernatant was counted in triplicate. The percentage of detached cell formation was then calculated by dividing the number of detached cells in the supernatant by the number of infected cells at 48 hpi.

### Immunofluorescence assay

3 × 10^4^ HeLa cells were seeded on coverslips in 24-well plates and infected the following day with *P. berghei* sporozoites. At 54 hpi, cells were fixed with 4% paraformaldehyde (PFA) in PBS for 20 minutes at room temperature, followed by permeabilization with ice-cold methanol. Sporozoites were isolated from salivary glands and allowed to adhere to glass coverslips for one hour at 37 °C. They were then similarly fixed with 4% PFA in PBS and permeabilized with ice-cold methanol as described before. Unspecific binding sites were blocked by incubation in 10% FCS in PBS, followed by incubation with primary antibodies (rabbit anti-GFP (Invitrogen, cat. no. A6455, 1:1000), mouse-anti-GFP (Roche, cat. no. 11814460001, 1:1000), rabbit-anti-CSP (Eurogentec, cat. no. A8240, 1:3000), chicken anti-EXP1 and rat anti-MSP1 (both generated at the Bernhard Nocht Institute (Hamburg, Germany), 1:250 and 1:1000) and subsequently with fluorescently labeled secondary antibodies (goat anti-rabbit AlexaFluor488 (Invitrogen, cat. no. A11034, 1:2000), goat anti-mouse AlexaFluor488 (Invitrogen, cat. no. A-11001, 1:2000), donkey anti-rat AlexaFluor488 (Molecular Probes, cat. no. A-21208, 1:2000), goat anti-rat AlexaFluor647 (Invitrogen, No. A21247, 1:2000) and donkey anti-chicken Cy5 (Dianova, cat. no. 703-175-155, 1:2000)) diluted in 10% FCS in PBS. DNA was visualized by staining with 1 µg/ml DAPI (Sigma). Labeled cells and sporozoites were mounted on microscope slides with Dako Fluorescent Mounting Medium (Dako) and analyzed by confocal point scanning microscopy using a Zeiss LSM5 Duo microscope and a Zeiss Plan-Apochromat 63×/1.4 oil objective. For staining of blood stage schizonts, coverslips in 24 well plates were coated with 40 μl of 0, 5 mg/ml concanavalin A (Sigma) in water for 30 minutes. Subsequently, 100 μl of infected red blood cells from a schizont culture were added to coverslips for 20 minutes and excess of blood was carefully removed. Cells were fixed with 4% PFA in PBS for 20 minutes and permeabilized with 0, 15% Triton-X-100 in PBS for 10 minutes at room temperature. Unspecific binding sites were blocked by incubation in 3% bovine serum albumin (BSA) in PBS, followed by incubation with primary and secondary antibodies (described above), which were diluted in 3% BSA in PBS. DNA was visualized by staining with 1 μg/ml DAPI. Labeled cells were mounted on microscope slides with Dako Fluorescent Mounting Medium and analyzed by using a Leica TCS SP8 confocal microscope with a HC PL 100x/1.40 oil objective. Image processing was performed using ImageJ.

### Generation of PbPMP1-KO parasites

PbPMP1-KO parasites were generated by transfection of the plasmoGEM vector pGEM-283874^38, 39^ into marker-free mCherry_*hsp70*_ parasites^17^ using standard methods of transfection^37^. Transgenic parasites were selected by pyrimethamine in the drinking water of infected mice and a clonal KO parasite line was generated by limiting dilution. Successful deletion of the PbPMP1 coding sequence was confirmed by diagnostic PCR as described above. All primer sequences are listed in Supplementary Table S1.

### Analysis of PbPMP1-KO parasites

Nine days after the infective blood meal, midguts of 15–23 mosquitoes were dissected into PBS and the pooled midguts were fixed in 4% PFA in PBS for 20 minutes at room temperature. The midguts were then washed with PBS and stored in PBS at 4 °C in the dark. The next day, fixed midguts were mounted on glass slides containing a small amount of Dako Fluorescent Mounting Medium and imaged using a fluorescence microscope with a 5x objective. The average number of oocysts per midgut was then determined using an ImageJ-based counting macro^40^. On day 18 after the infective blood meal, salivary glands of 10 mosquitoes were isolated, homogenized and released sporozoites were counted using a hemocytometer.

For the *in vivo* analysis of PbPMP1-KO parasites, 1,000 WT or PbPMP1-KO sporozoites were injected intravenously into four C57BL/6 mice per group. Subsequently, blood stage parasitemia was determined between day 3 and 6 post-infection by FACS analysis using a FACSCalibur flow cytometer (BD Biosciences) and the mCherry fluorescence of parasites.

For the determination of sporozoite infectivity, 5 × 10^4^ HeLa cells per well were seeded in 24-well plates and infected the next day with 10,000 WT and PbPMP1-KO sporozoites. After 48 hpi, the average number of infected host cells per well was quantified in triplicate. Size measurement and determination of detached cell numbers were performed as described above.

### Live-cell imaging of transgenic parasite lines

Mixed blood stages, cultured schizonts and ookinetes were imaged on a Leica DM5500 B epifluorescence microscope using a HCX Plan-Apochromat 100x/1.4 oil objective and for visualization of DNA, 1 µg/ml Hoechst 33342 (Sigma) was added. Resulting images were deconvoluted using Huygens software (Scientific Volume Imaging). For imaging of oocysts, midguts of infected mosquitoes were isolated at different days after the infectious blood meal and placed into PBS containing 10 µg/ml Hoechst 33342. Oocysts were subsequently analyzed by confocal line scanning using a Zeiss LSM5 Duo microscope and a Zeiss Plan-Apochromat 63x/1.4 oil objective or by confocal point scanning using a Leica TCS SP8 confocal microscope with a HC PL APO 63x/1.40 oil objective.

For imaging of liver stage parasites, 1 × 10^5^ HeLa cells were seeded into live-cell imaging dishes (MatTek, *In Vitro* Scientific) and infected the next day with transgenic sporozoites. At different time points post-infection, 1 µg/ml Hoechst 33342 was added to the culture medium and parasites were analyzed by confocal point scanning microscopy using the Zeiss LSM5 Duo microscope or the Leica TCS SP8 as described above. For live-cell time-lapse microscopy, HeLa cells were infected with transgenic sporozoites as already described and parasite development was followed for 12 hours using the Zeiss LSM5 Duo microscope in the LIVE mode (confocal line scanning). Imaging was started at 48 hpi and every 10 minutes an image was acquired by the Zeiss LSM Multitime-Macro. During imaging, infected HeLa cells were kept in a CO_2_ incubator at 37 °C. Image processing was performed using ImageJ.

### FLIP experiments

FLIP experiments were performed on a TILL Photonics iMIC equipped with a 488-nm diode laser and an Olympus UPlanSApo 60x/1.35 oil objective. Images were taken using a confocal spinning disk system. For each FLIP experiment, an image was acquired before photobleaching, followed by 40 cycles of bleaching with five seconds delay between cycles. In each cycle, a region of interest was photobleached with high laser power and afterwards an image was acquired with low laser power. Fluorescence intensity values were calculated from background-subtracted images and the fluorescence intensity before bleaching was set to 100%. During imaging, cells were kept in a CO_2_ incubator at 37 °C. Image processing was performed using ImageJ.

### Serial block face scanning electron microscopy

HeLa cells (5 × 10^4^) were seeded per well into a 96-well plate and infected with mCherry_*hsp70*_ parasites^17^. Infected cells were FACS-sorted at 6 hpi and re-seeded at a density of 10,000 sorted cells per well into a 96-well optical plate. Following this, parasites were fixed in a glutaraldehyde buffer at 48 hpi and processed according to a previously published protocol^41^. SBFSEM images were acquired with a Quanta FEG 250 (FEI Company) equipped with a Gatan 3View2XP ultramicrotome (accelerating voltage 5 3.5 kV; low vacuum). Image processing was performed using ImageJ.

### Dual color STED super-resolution microsopy

HeLa cells infected with sfGFP-PbSec61β/PbPMP1-mC parasites were fixed at 48 hpi, permeabilized and stained with first antibodies (mouse anti-mCherry (abcam, cat. no. ab125096, 1:100), rabbit anti-GFP (Invitrogen, cat. no. A6455, 1:100)) and secondary antibodies (goat anti-mouse-AlexaFluor594 (Molecular Probes, cat. no. A11032, 1:200), goat anti-rabbit-ATTO647N (Sigma, cat. no. 40839, 1:200)) as described above. Coverslips were then embedded in Mowiol (Roth) containing 2.5% DABCO (Roth) antifade. Dual color STED microscopy was performed on a Leica TCS SP8 confocal microscope equipped with a HC PL APO 100x/1.40 oil objective, a white light excitation laser and a 775-nm pulsed depletion laser. Image processing was performed using ImageJ.

### Statistical analyses

Statistical analyses were performed using GraphPad Prism (GraphPad Software). For comparisons between two groups, an unpaired two-tailed t-test was used. For comparison between three groups, a one-way ANOVA followed by a Holm-Sidak multiple comparison test was performed. P values of < 0.05 were considered significant.

### Data Availability

All data generated or analyzed during this study are included in this published article (and its Supplementary Information files).

## Electronic supplementary material

Supplementary Information

Supplementary Movie S1

Supplementary Movie S2

Supplementary Movie S3

Supplementary Movie S4

Supplementary Movie S5

Supplementary Movie S6
