# Supplementary material for: A Plasmodium plasma membrane reporter reveals membrane dynamics by live-cell microscopy
Source: Sci Rep. 2017 Aug 29;7:9740. doi: 10.1038/s41598-017-09569-4 (PMC5575152; doi:10.1038/s41598-017-09569-4)
Supplement: Supplementary file 1 — Supplementary Information [file 41598_2017_9569_MOESM1_ESM.pdf]

Supplementary Information

A *Plasmodium* plasma membrane reporter reveals membrane dynamics by live-cell microscopy

Paul-Christian Burda[1,#,*], Marco Schaffner[1], Gesine Kaiser[1], Magali Roques[1], Benoît Zuber[2], Volker T. Heussler[1]

[1]Institute of Cell Biology, University of Bern, 3012 Bern, Switzerland
[2]Institute of Anatomy, University of Bern, 3012 Bern, Switzerland

[#]Present address: Bernhard Nocht Institute for Tropical Medicine, 20359 Hamburg, Germany
*Corresponding author

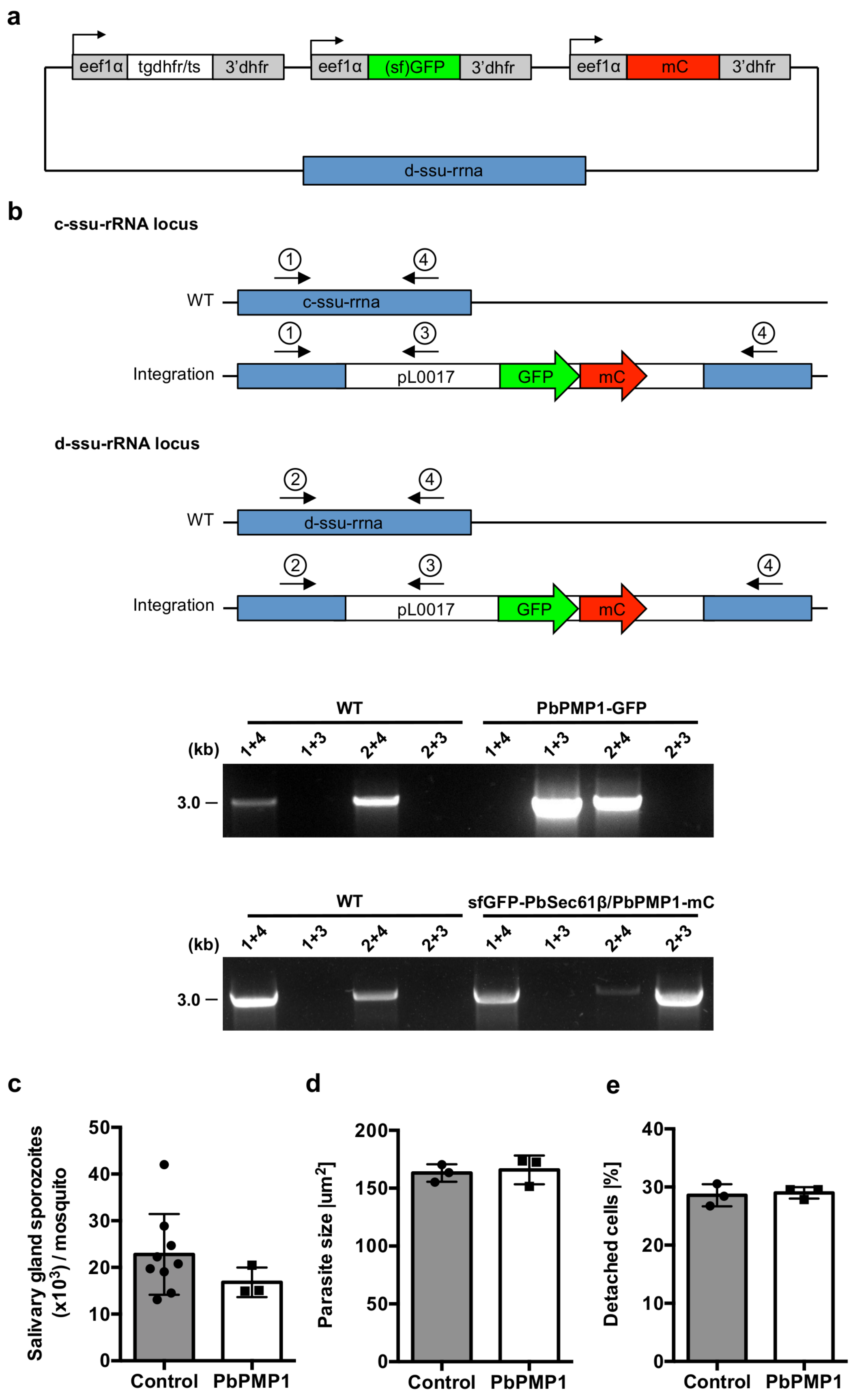

a
eef1α
tgdhfr/ts
3'dhfr
eef1α
(sf)GFP
3'dhfr
eef1α
mC
3'dhfr
d-ssu-rrna
b
c-ssu-rRNA locus
WT
c-ssu-rrna
Integration
pL0017
GFP
mC
d-ssu-rRNA locus
WT
d-ssu-rrna
Integration
pL0017
GFP
mC
WT
PbPMP1-GFP
(kb)
1+4
1+3
2+4
2+3
3.0
WT
sfGFP-PbSec61β/PbPMP1-mC
(kb)
3.0
c
Salivary gland sporozoites (x10³) / mosquito
Control
PbPMP1
d
Parasite size [um²]
Control
PbPMP1
e
Detached cells [%]
Control
PbPMP1

Supplementary Figure S1. Integration PCR of reporter parasite lines and characterization of PbPMP1-GFP parasites (related to Fig. 1 and Fig. 6). (a) Schematic representation of the pL0017 based plasmids used to generate PbPMP1-GFP and sfGFP-PbSec61β/PbPMP1-mCherry parasites. This vector integrates into the *c-* or the *d-ssu-rRNA* locus via single crossover recombination and conveys resistance to pyrimethamine. **(b)** Diagnostic PCR of PbPMP1-GFP and sfGFP-PbSec61β/PbPMP1-mCherry parasites. Successful integration of the transfected plasmids into either of two possible loci in the *P. berghei* genome was tested by PCR. Locations of primers used for PCR analysis are shown. For each locus, one primer pair (1 and 4, 2 and 4, respectively) yields a PCR product of 3 kb if no integration has taken place. In the case of successful integration, the primers are too far apart (>14 kb) to result in a complete PCR product under the chosen conditions. To further confirm integration, additional primer pairs (1 and 3, 2 and 3) were used, which only generate a PCR product of 3 kb if the plasmid has integrated. All primer sequences are listed in Supplementary Table S1. **(c)** PbPMP1-GFP parasites show normal numbers of sporozoites in the salivary glands. 18 days after the infectious blood meal, the average number of sporozoites in the mosquito salivary glands was determined from 10 mosquitoes in each experiment. Shown are means with SD of three (PbPMP1-GFP) to nine (control) independent mosquito feed experiments. **(d)** PbPMP1-GFP parasites develop normally in size. HeLa cells were infected with control and PbPMP1-GFP parasites and 48 hpi parasite size (area) was determined by density slicing using ImageJ. For each parasite line, the average size of 50-100 parasites was determined in each experiment. **(e)** PbPM1-GFP parasites do not differ in their capacity to produce detached cells. Detached cells in the supernatant were counted at 65 hpi in triplicate and were normalized to the number of infected cells at 48 hpi. Shown are means with SD of three independent experiments. For statistical analysis, a two-tailed unpaired t-test was performed, which did not detect significant differences in any of the assays ($p > 0.05$).

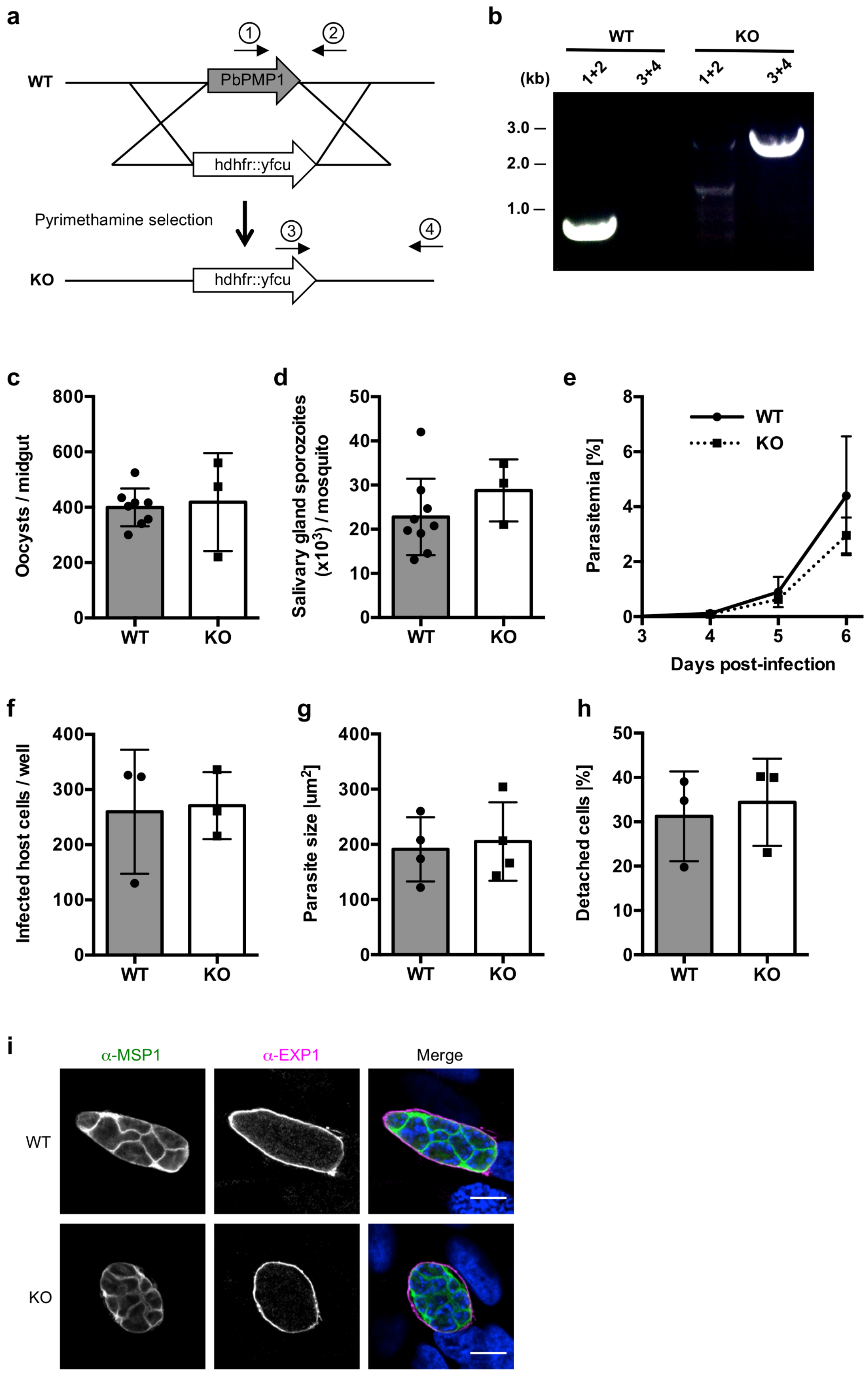
a
WT
PbPMP1
hdhfr::yfcu
Pyrimethamine selection
KO
hdhfr::yfcu
b
WT
KO
(kb)
1+2
3+4
1+2
3+4
3.0
2.0
1.0
c
Oocysts / midgut
WT
KO
d
Salivary gland sporozoites (x10³) / mosquito
WT
KO
e
Parasitemia [%]
WT
KO
Days post-infection
f
Infected host cells / well
WT
KO
g
Parasite size [um²]
WT
KO
h
Detached cells [%]
WT
KO
i
α-MSP1
α-EXP1
Merge
WT
KO

Supplementary Figure S2. Generation and characterization of PbPMP1-knockout parasites. (a) Schematic representation of the knockout (KO) strategy. A clonal PbPMP1-KO parasite line was generated by transfection of wild-type (WT) blood stage parasites with a plasmoGEM vector containing a fusion of the positive drug selectable marker *hdhfr* (human dihydrofolate reductase) and the negative marker *yfcu* (yeast cytosine deaminase and uridyl phosphoribosyl transferase) under the control of the *eef1α* promoter targeting the PbPMP1 coding sequence by double crossover homologous recombination followed by pyrimethamine selection. Locations of primers used for PCR analysis are shown. **(b)** Diagnostic PCR to confirm PbPMP1-KO parasites. Primer 1 and 2 are expected to give a product of 691 bp in the case of WT parasites, while primer 3 and 4 are expected to yield a product of 2360 bp for KO parasites. All primer sequences are listed in Supplementary Table S1. **(c)** PbPMP1-KO parasites produce normal numbers of oocysts in comparison to WT parasites. Nine days after the infectious blood meal, midguts were removed and the average number of oocysts per midgut was determined from 15-23 mosquitoes per experiment. **(d)** PbPMP1-KO parasites show normal numbers of sporozoites in the salivary glands. 18 days after the infectious blood meal, the average number of sporozoites in the mosquito salivary glands was determined from 10 mosquitoes in each experiment. Shown are means with SD of three (PbPMP1-KO) to nine (WT) independent mosquito feed experiments. **(e)** PbPMP1-KO parasites develop normal blood stage parasitemia after sporozoite infection. Four C57BL/6 mice per group were injected intravenously with 1,000 WT or PbPMP1-KO sporozoites and subsequent blood stage parasitemia was measured by FACS analysis between day 3 and 6 post-infection (shown as means with SD). In all WT and KO-infected mice, the first parasites were detected in the blood three days after sporozoite infection and there was no statistically significant difference in blood stage parasitemia between the groups (unpaired two-tailed t-test, $p > 0.05$), indicating normal liver stage and blood stage development *in vivo*. **(f)** PbPMP1-KO sporozoites have a similar infectivity as WT sporozoites. HeLa cells were infected with 10,000 WT or KO sporozoites and the average number of infected host cells per well was quantified 48 hpi in triplicate. **(g)** PbPMP1-KO parasites develop normally in size. HeLa cells were infected with WT and KO parasites and 48 hpi, parasite size (area) was determined by density slicing using ImageJ. For each parasite line, the average size of 50-100 parasites was determined in each experiment. **(h)** PbPMP1-KO parasites do not differ in their capacity to produce detached cells. Detached cells in the supernatant were counted at 65 hpi in triplicate and were normalized to the number of infected cells at 48 hpi. **(i)** PbPMP1-KO liver stage parasites show normal MSP1 and EXP1 expression and localization. HeLa cells were infected with WT and KO sporozoites, fixed at 54 hpi and IFA was performed using an antiserum against the PPM marker protein MSP1 (green) and the PVM marker protein EXP1 (purple). The merged channels additionally contain DAPI-stained nuclei (blue). Scale bars = 10 μm. If not stated otherwise, for all counting experiments means with SD of three to four independent experiments are shown. For statistical analysis, a two-tailed unpaired t-test was performed, which did not detect significant differences in any of the assays ($p > 0.05$).

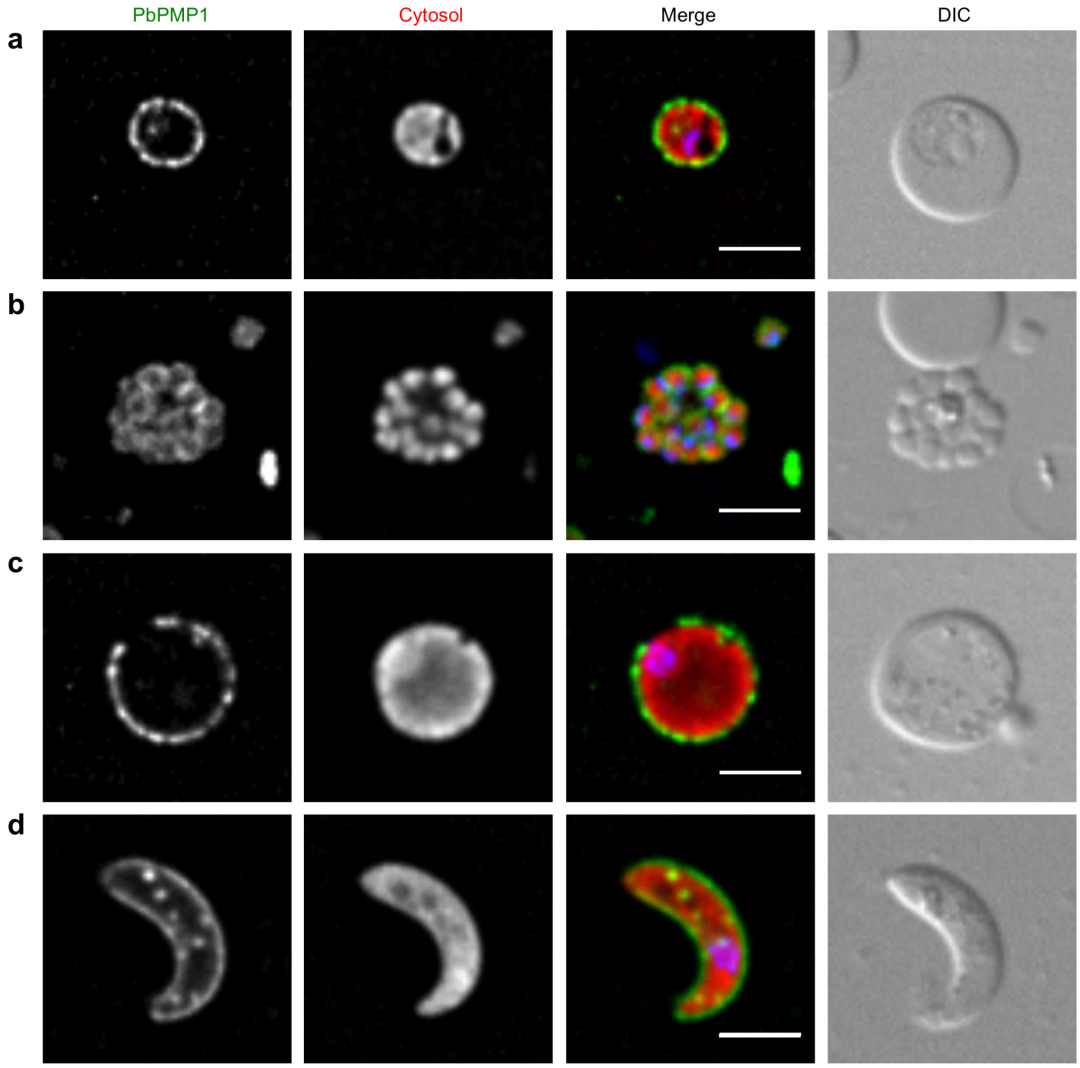

Supplementary Figure S3. Plasma membrane morphology in erythrocytic asexual and sexual blood stages and in ookinetes. The localization of PbPMP1-GFP in live parasites was analyzed by epifluorescence microscopy. **(a)** Trophozoite. **(b)** Merozoites. **(c)** Gametocyte. **(d)** Ookinete, which was obtained by dissecting mosquito midgut content 24 hours after the infectious blood meal. PbPMP1-GFP (green). Parasite cytosol (red). DNA was stained with Hoechst 33342 (blue) and is displayed in the merged channels. DIC = Differential interference contrast. Scale bars = 5 µm.

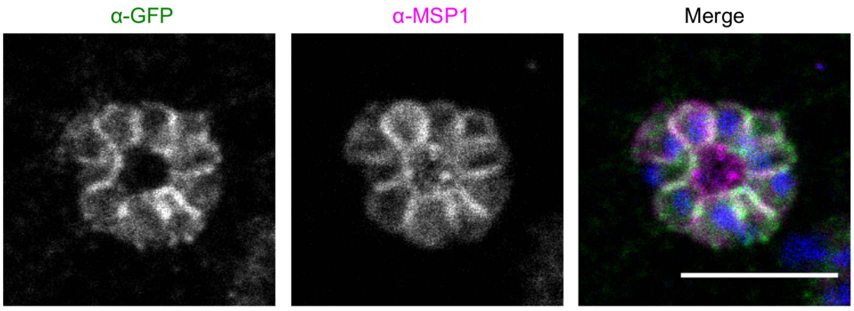

Supplementary Figure S4. PbPMP1-GFP co-localizes with the plasma membrane marker MSP1 in blood stage schizonts. Cultured schizonts were fixed and IFA was performed using an antiserum against the PPM marker MSP1 (purple). The PbPMP1-GFP signal was enhanced by staining with an anti-GFP antiserum (green). The merged channel additionally contains DAPI-stained nuclei (blue). Scale bar = 5 μm.

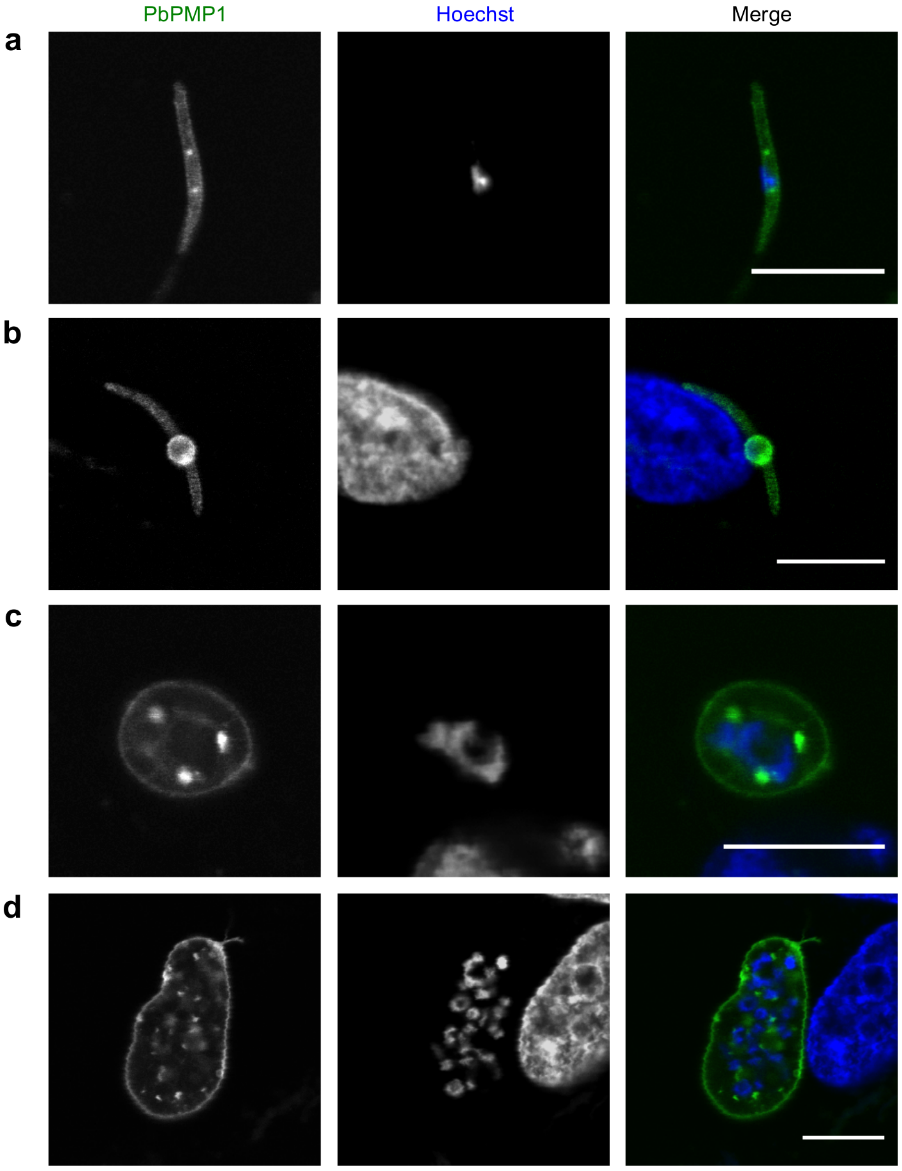

Supplementary Figure S5. Plasma membrane morphology of live PbPMP1-GFP sporozoites and early liver stage parasites (related to Fig. 3). (a) PbPMP1-GFP sporozoites were isolated from the salivary glands of infected mosquitoes and imaged by confocal microscopy. **(b)**, **(c)** and **(d)** HeLa cells were infected with PbPMP1-GFP sporozoites and analyzed by confocal microscopy at 5 (b), 24 (c) and 30 (d) hpi. PbPMP1-GFP (green). DNA was stained with Hoechst 33342 (blue). Scale bars = 10 µm.

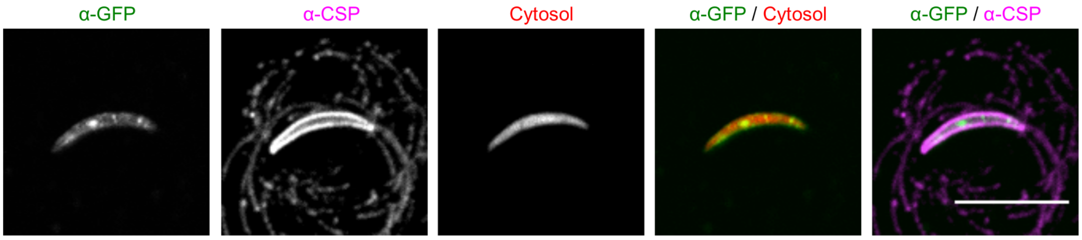

Supplementary Figure S6. PbPMP1-GFP partially co-localizes with CSP. Salivary gland sporozoites were fixed and IFA was performed using an antiserum against CSP (purple). The PbPMP1-GFP signal was enhanced by staining with an anti-GFP antiserum (green). Parasite cytosol (red). Scale bar = 10 µm.

Supplementary Movie S1. Confocal z-stack images of PbPMP1-GFP oocysts at the time of plasma membrane invagination (related to Fig. 2c). Midguts of PbPMP1-GFP parasite-infected mosquitoes were isolated at day 11 after the infectious blood meal and z-stacks were taken by confocal microscopy. PbPMP1-GFP (green). DNA was stained with Hoechst 33342 (blue). Scale bar = 10 µm.

Supplementary Movie S2. Confocal z-stack images of PbPMP1-GFP oocysts with completely formed sporozoites (related to Fig. 2e). Midguts of PbPMP1-GFP parasite-infected mosquitoes were isolated at day 11 after the infectious blood meal and z-stacks were taken by confocal microscopy. PbPMP1-GFP (green). DNA was stained with Hoechst 33342 (blue). Scale bar = 10 µm.

Supplementary Movie S3. Visualization of PPM dynamics during late liver stage development by live-cell time-lapse microscopy (related to Fig. 4). HeLa cells were infected with PbPMP1-GFP parasites and live-cell imaging was started at 48 hpi. Images were taken every 10 minutes using a confocal microscope. 4 frames per second. Scale bar = 10 µm.

Supplementary Movie S4. Confocal z-stack images of an sfGFP-PbSec61β/PbPMP1-mCherry oocyst (related to Fig. 6c). Midguts of sfGFP-PbSec61β/PbPMP1-mCherry parasite-infected mosquitoes were isolated at day 7 after the infectious blood meal and z-stacks were taken by confocal microscopy. PbSec61β (green). PbPMP1-mCherry (red). Scale bar = 10 µm.

Supplementary Movie S5. Confocal z-stack images of an sfGFP-PbSec61β/PbPMP1-mCherry liver stage parasite (related to Fig. 6d).
HeLa cells were infected with sfGFP-PbSec61β/PbPMP1-mCherry parasites and z-stacks were taken by confocal microscopy at 48 hpi. PbSec61β (green). PbPMP1-mCherry (red). Scale bar = 10 µm.

Supplementary Movie S6. Visualization of ER/PPM interactions by live-cell time-lapse microscopy (related to Fig. 6). HeLa cells were infected with sfGFP-PbSec61β/PbPMP1-mCherry parasites and live-cell imaging was started at 48 hpi. Images were taken every 10 minutes using a confocal microscope. 4 frames per second. Scale bar = 10 µm.

Supplementary Table S1. Primers used in this study. Restriction sites are underlined if present.

Name	Lab-No.	Sequence (5'-3')	Purpose
PbPMP1-GFP-fw	417	GCGGATCCATGACTATAAATATAAGA TGGAGTAG	Generation of PbPMP1-GFP parasites (Fig. 1)
PbPMP1-GFP-rev	418	GCGGATCCATTGGATTTATTCTTTAG CTCAACTTC	Generation of PbPMP1-GFP parasites (Fig. 1)
eef1a-fw	41	CGTAGGTACCAGCTTAATTCTTTTCG AGCTCTTT	Generation of PbPMP1-GFP (Fig. 1) and sfGFP-PbSec61β/PbPMP1-mCherry parasites (Fig. 6)
3dhfr-rev	42	ACTGGGTACCCGAAATTGAAGGAAAA AACATCATTTG	Generation of PbPMP1-GFP (Fig. 1) and sfGFP-PbSec61β/PbPMP1-mCherry parasites (Fig. 6)
Primer-1	728	GTGTAGTAACATCAGTTATTGTGTG	Diagnostic PCR of PbPMP1-GFP and sfGFP-PbSec61β/PbPMP1-mCherry parasites (Supplementary Fig. S1)
Primer-2	729	ATACTGTATAACAGGTAAGCTGTTAT TGTG	Diagnostic PCR of PbPMP1-GFP and sfGFP-PbSec61β/PbPMP1-mCherry parasites (Supplementary Fig. S1)
Primer-3	730	TTTCCCAGTCACGACGTTG	Diagnostic PCR of PbPMP1-GFP and sfGFP-PbSec61β/PbPMP1-mCherry parasites (Supplementary Fig. S1)
Primer-4	731	CTTAGTGTTTTGTATTAATGTCGATTT G	Diagnostic PCR of PbPMP1-GFP and sfGFP-PbSec61β/PbPMP1-mCherry parasites (Supplementary Fig. S1)
Primer-1	430	TCTTGCCTTGGTTGCACACA	Diagnostic PCR of WT and PbPMP1-KO parasites (Supplementary Fig. S2)
Primer-2	431	ACAATTGAGGAATGCTAGCGT	Diagnostic PCR of WT and PbPMP1-KO parasites (Supplementary Fig. S2)
Primer-3	420	CTTTGGTGACAGATACTAC	Diagnostic PCR of WT and PbPMP1-KO parasites (Supplementary Fig. S2)
Primer-4	432	GCTGGTTCCTCCGTAAATTCAGCCC	Diagnostic PCR of WT and PbPMP1-KO parasites (Supplementary Fig. S2)
